# Identification of Psychological Factors Associated with Adherence to Self-Care Behaviors amongst Patients with Type 1 Diabetes

**DOI:** 10.1155/2019/6271591

**Published:** 2019-08-14

**Authors:** Dácil Alvarado-Martel, M. Ángeles Ruiz Fernández, Maribel Cuadrado Vigaray, Armando Carrillo, Mauro Boronat, Ana Expósito Montesdeoca, Ana M. Wägner

**Affiliations:** ^1^Department of Endocrinology and Nutrition, Complejo Hospitalario Universitario Insular Materno-Infantil de Gran Canaria, Las Palmas de Gran Canaria, Spain; ^2^Instituto Universitario de Investigaciones Biomédicas y Sanitarias, Universidad de Las Palmas de Gran Canaria, Las Palmas de Gran Canaria, Spain; ^3^Universidad Nacional de Educación a Distancia (UNED), Madrid, Spain; ^4^Department of Endocrinology and Nutrition, CIBER of Diabetes and Associated Metabolic Diseases, Hospital Universitario Germans Trias i Pujol, Badalona, Spain

## Abstract

**Purpose:**

To explore the factors involved in adherence to self-care behaviors in patients with type 1 diabetes.

**Materials and Methods:**

Patients with type 1 diabetes (age range: 14-71 years) were invited to participate at seven Spanish hospitals. They completed a dossier which recorded sociodemographic and clinical variables and also measured personality variables, emotional state, beliefs, and concerns regarding the illness, by means of questionnaires.

**Results:**

A total of 428 patients with type 1 diabetes were included (58% women, age 36 (11.8) years, diabetes duration 18.3 (10.2) years, HbA1c 7.9 +/-1.3%). A total of 60.1% of patients found it difficult to follow the treatment recommendations for the care of their disease. The reasons given were mood (25.2%), lack of motivation (13.4%), work (12%), and economic difficulties (3.8%). Other personal reasons were reported by 5.7%. Motivation, training in diabetes management, importance the patient attributed to the disease, and self-efficacy were the variables that predicted adherence to self-care behaviors, together accounting for 32% of its variance. Anxiety and depression were highly prevalent in this study population (57.1% and 23.1%, respectively) and were associated with lower adherence.

**Conclusion:**

In the present study assessing patients with type 1 diabetes, motivation, training in diabetes management, beliefs regarding the disease, and self-efficacy were the main contributors to adherence to self-care behaviors. On the other hand, anxiety and depression were highly prevalent and associated with lower adherence. Thus, supplementing therapeutic education with strategies designed to raise levels of motivation, discussion of beliefs about the disease, and encouragement of self-efficacy might be a useful way to increase patient involvement in self-care.

## 1. Introduction

Type 1 diabetes is a difficult disease to treat successfully, as even the most motivated patients find it hard to meet all the demands of self-management. The numerous finger pricks to measure glucose levels, the multiple insulin injections, the need to count carbohydrates in the diet, exercise, hypoglycemia prevention and treatment, and the constant need to make decisions regarding dose calculation require a high level of commitment from patients every day of their lives [1, 2]. The goal in diabetes treatment is to achieve a balance: that is, to perform daily self-care tasks adequately in order to optimize glycemic control without allowing the condition to interfere with and limit the patient's everyday life [3].

In recent years, many of the therapeutic advances have indeed focused on making it easier for patients to live with diabetes. Some examples of these are the emergence of new technologies, the development of drugs with lower risk of hypoglycemia, and educational programs that promote a flexible diet [4]. Despite the improvements in the level of patient education, however, adherence remains low [5], especially with regard to recommendations that require lifestyle changes, such as glucose monitoring and carbohydrate counting [6].

Adherence is a dynamic, multidimensional process in which many factors play a part [7]. Demographic, psychological, and social factors have been associated with adherence in patients with diabetes [8]. Therapeutic education has also proven to be an essential component of adherence to self-care [9], but that alone does not guarantee patients' full involvement; they may internalize therapeutic recommendations but will then decide whether to adhere to them or not. For all these reasons, it is important to be able to implement strategies that support behavior change [10].

The concept of adherence has been widely discussed in the literature, but the research has focused mainly on single interventions, such as insulin therapy [11] or diet [12], or selected associated variables, such as personality, beliefs [13], self-efficacy, and social support [14]. Indeed, there are many factors that can, directly or indirectly, influence disease self-management [15, 16]. To our knowledge, there are no previous studies assessing adherence to self-care in type 1 diabetes with a multifactorial approach.

The aim of the present study was to identify factors that may promote or hinder adherence to self-care behaviors in patients with type 1 diabetes. The information obtained should help in the design of strategies to improve patients' engagement with their treatment.

## 2. Materials and Methods

### 2.1. Participants and Procedure

This study was part of a cross-sectional study in 7 hospitals in Spain: Complejo Hospitalario Universitario Insular Materno-Infantil de Gran Canaria, Hospital Universitario de Gran Canaria Doctor Negrín, Hospital Universitario Germans Trias i Pujol in Badalona, Hospital Universitario Ramón y Cajal in Madrid, Hospital Universitario La Paz in Madrid, Hospital Universitario Parc Tauli in Sabadell, and the private clinic D-Médical, Madrid.

Patients diagnosed with type 1 diabetes and attending outpatient clinics for routine visits in the Endocrinology and Nutrition Services at the mentioned hospitals in Spain were invited to participate, between November 2014 and April 2016. The research project was led by the Complejo Hospitalario Universitario Insular Materno-Infantil de Gran Canaria, where most of the sample was recruited.

To assure representativeness, several weekdays were selected before the start of the study, and all patients attending their routine visits those days (and followed by different physicians) were invited to participate.

Patients aged < 14 years, pregnant women, and people who could not complete the dossier due to language problems were excluded from the study.

Patients were previously informed of the purpose of the study and of the voluntary nature of participation and were assured that the information provided would be treated confidentially. They were given an informed consent form to complete, as well as a dossier containing questions regarding their sociodemographic and biomedical data and the questionnaires to measure psychosocial variables. Patients completed the questionnaires in the waiting room, and a researcher was available at all times to answer questions. This study is part of a broader research project aimed at validating a health-related quality of life questionnaire named ViDa1, specifically designed by the authors to measure health-related quality of life in patients with type 1 diabetes [17]. The study was approved by the hospitals' Ethics Committees.

## 3. Measures

### 3.1. Dependent Variable

#### 3.1.1. Adherence to Self-Care Behaviors

The validated Spanish version of the Diabetes Self-Care Inventory-Revised version (SCI-R) [18] was used. This inventory consists of 15 items that refer to self-care behaviors in the treatment of diabetes, which are scored on a Likert scale ranging from 1 = “never” to 5 = “always.” The scores are converted with a formula, and the responses range from 0 to 100; higher scores indicate higher levels of self-care.

### 3.2. Independent Variables

#### 3.2.1. Sociodemographic and Biomedical Variables

A data sheet was designed specifically for the study, and it covered the following sociodemographic and clinical variables: sex, age, level of education (illiterate, primary, secondary, and university studies), employment situation, living arrangements, duration of disease, type of drug treatment, glycemic control (the most recent glycosylated hemoglobin (HbA1c) standardized against NGSP/DCCT), treatment with psychoactive drugs, cardiovascular risk factors (diagnosis of hypertension, dyslipidemia, smoking, and obesity), carbohydrate count, presence and type of chronic complications and the limitation they represented on participants' daily lives, number of hypoglycemic episodes per week, and presence of acute complications (admissions for severe hyperglycemia or hypoglycemia). The medical variables were confirmed with the patient's medical history.

#### 3.2.2. Level of Motivation and Barriers to Self-Care

Level of motivation and barriers to self-care were measured through the responses to the following item on the data collection sheet: “Do you find it hard to follow the treatment recommended for your diabetes?” Participants who answered yes were asked to identify the barriers to self-care from amongst several possible answers: economic difficulties, mood, work, lack of motivation, not worth the effort, and other unidentified barriers. Motivation was measured through the responses to the item “My level of motivation for self-managing my diabetes” on a Likert scale (1 to 10).

#### 3.2.3. Patients' Perceptions of Their Knowledge of Diabetes

Patients' perceptions of their knowledge of diabetes were measured from their responses to the following item on the data collection sheet: “How would you rate your level of training in diabetes management?” The answers were rated on a Likert scale (1-10), higher scores implying better (self-perceived) training. In addition, the following question was made: “Does your level of training in diabetes make management of the disease easier?” where the reply was yes or no.

#### 3.2.4. Beliefs about the Disease

Beliefs about the disease were measured assessing three aspects: level of disease awareness (with the question “To what extent are you aware of the type of disease you have?”); level of importance given to the disease (“Please score the importance you give the disease”); and level of severity perceived (“Please score the severity to attribute to the disease”). All three issues were rated on a Likert scale (1-10), higher scores reflecting higher awareness, importance, and severity, respectively.

#### 3.2.5. Concern about Developing Complications in the Future

The concern about developing complications in the future was measured with the item “I'm worried about developing chronic complications of diabetes in the future” (ViDa1) [17] on a Likert scale ranging from 1 = “strongly disagree” to 5 = “strongly agree.” To calculate the percentage of patients who were worried, those who answered “strongly agree” (5) or “agree” (4) were considered.

#### 3.2.6. Health-Related Quality of Life

Health-related quality of life was measured with ViDa1 [17], which contains 34 items grouped in four dimensions: interference of diabetes in daily life, self-care, well-being, and worry about the disease. The response format is a Likert scale ranging from 1 = “strongly disagree” to 5 = “strongly agree.” A total score is obtained for each subscale, a higher value indicating a higher level of the respective aspect.

#### 3.2.7. Personality

*(i) Self-Efficacy*. Self-efficacy was measured by the Spanish version of the General Self-Efficacy Scale (GSE) [19]. This scale measures respondents' expectations about their ability to cope adequately with a problematic situation. Responses are recorded on a Likert scale (1 = “not at all” to 5 = “totally”), and the score range is 1–50. High scores indicate a higher perception of self-efficacy.

*(ii) Affectivity*. Affectivity was measured through a shortened version of the Positive and Negative Affect Schedule (PANAS) [20]. Positive affect (PA) represents the dimension of pleasant emotions, reflecting the degree to which a person feels enthusiastic, motivated, active, energetic, and so on. It is related to extroversion and optimism. Negative affect (NA) represents the dimension of unpleasant emotions, a variety of aversive emotional states such as fear, inhibitions, insecurities, frustration, and failure. People with high NA often experience lack of interest, sadness, guilt, anger, fear, shame, anxiety, and envy; NA is related to pessimism, stress, dissatisfaction, and negative self-appraisal. The PANAS has 10 items, five evaluating NA and five PA. The response format is a Likert scale ranging from 0 = “not at all” to 6 = “completely” with an overall score ranging from 0 to 30 for each subscale.

*(iii) Conscientiousness*. Conscientiousness was measured using the Spanish version of the subscale of the Big Five Inventory (BFI-35) [21]. People with high scores on this dimension are organized, self-disciplined, and perseverant. It consists of seven items scored on a Likert scale (1 = “strongly disagree” to 5 = “strongly agree”) with an overall score ranging between 7 and 35.

#### 3.2.8. Emotional State

*(i) Symptoms of Anxiety and Depression*. Symptoms of anxiety and depression were measured with the Spanish version of the Hospital Anxiety and Depression Scale (HADS) [22]. This 14-item scale assesses seven symptoms of anxiety and seven of depression. The response format is a Likert scale (0-3) and the score range for each subscale is 0-21. The score was interpreted using the cutoffs established by the authors. Scores of 0-7 indicate normality, 8-10 a probable case, and 11-21 a confirmed clinical case. In this study, we used the cutoff point of 8.

*(ii) Diabetes-Related Distress*. The diabetes-related distress was measured with the Spanish version of the Problem Areas in Diabetes Scale (PAID) [23]. The PAID consists of 20 items scored on a Likert scale (0 = “not a problem” to 4 = “a very serious problem”). The scores are added together and multiplied by 1.25, generating a total score ranging from 0 to 100. We used the cutoff set by the author [24].

*(iii) Fear and Concern regarding Hypoglycemia*. Fear and concern regarding hypoglycemia were measured with two items “I'm afraid of having hypoglycemias” and “I'm worried about having hypoglycemias” (ViDa1) [17] on a Likert scale ranging from 1 = “strongly disagree” to 5 = “strongly agree.” To calculate the percentage of patients who were afraid or worried, those who answered “strongly agree” (5) or “agree” (4) were considered.

#### 3.2.9. Social Support

Social support was measured with the Spanish version of the MOS-SSS Social Support Questionnaire [25]. This questionnaire has 20 items; the first has an individual interpretation and the other 19 are measured on a Likert scale and scored from 1 = “never” to 5 = “always.” The score ranges from 19 to 95, with higher scores indicating higher levels of perceived social support.

## 4. Statistical Analysis

Data were analyzed using SPSS version 23.0. (IBM Corp., Armonk, NY). All the variables studied were normally distributed according to the Kolmogorov-Smirnov test. Descriptives were calculated for all quantitative variables (mean and standard deviation) and percentages and frequencies for qualitative variables. Correlations were performed using the Pearson correlation coefficient. For multiple comparisons, the Bonferroni correction was applied to control for the probability of a type I error. Student's *t*-test and ANOVA were used to analyze the differences between groups. The predictors of adherence to self-care behaviors were analyzed using stepwise multiple linear regression. Adherence to self-care behaviors on the SCI-R was established as the dependent variable, and independent variables were those related to beliefs regarding the disease, personality, emotional state, and training in disease management, in order to identify the ones that require intervention to complement therapeutic education: i.e., self-perceived level of training, disease awareness, the importance the patient attributes to the disease, motivation for self-care, PA, self-efficacy, conscientiousness, anxiety, depression, and NA. To assess collinearity, we calculated the correlation matrix and the variance inflation factor (VIF) of each variable.

## 5. Results

A total of 428 people with type 1 diabetes aged between 14 and 71 years were enrolled at seven Spanish hospitals. The characteristics of the sample are shown in Table 1.

Table 2 displays the mean scores, standard deviations, and ranges of scores for each of the aspects measured and shows that the self-perceived level of training was high: 92.7% of respondents answered “yes” to the question of whether knowledge about diabetes facilitated self-care.

The level of motivation for self-care had a mean score below 7. A total of 60.1% of patients found it difficult to follow the treatment recommendations for the care of their disease. The reasons given were mood (25.2%), lack of motivation (13.4%), work (12%), and economic difficulties (3.8%). Other personal reasons were reported by 5.7%.

### 5.1. Emotional State

With regard to emotional state, the prevalence of anxiety was 57.1% (32.3% in probable cases and 24.8% in clinical cases) and that of depression 23.1% (14.6% in probable cases and 8.5% in clinical cases). According to the cutoff point established by the PAID, 48.1% of patients suffered distress related to diabetes.

Fear of hypoglycemia was reported by 56.8% of patients and frequent concern about the possibility of hypoglycemia by 64.4%. A total of 80.7% of patients expressed concern about developing chronic complications of diabetes later in life.

### 5.2. Relationship between Adherence to Self-Care (SCI-R) and the Study Variables

People who reported having difficulty following treatment recommendations had lower scores on the SCI-R (61.4 ± 15.1 vs. 72.3 ± 11 (*t* = 8.2; *p* < .001)), and those who stated that the level of training in diabetes management enhanced their self-care scored higher (66.5 ± 13.8 vs. 51.3 ± 20.8 (*t* = −5.5; *p* < .001)).

**Figure 1:**
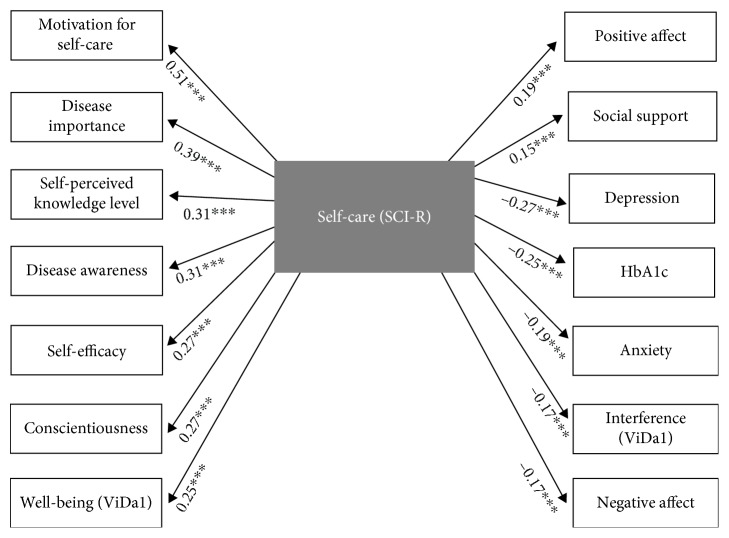
Significant correlations between adherence to self-care and the rest of the variables. *N* = 428; ^∗∗∗^*p* < .001. Bonferroni correction was applied.

Adherence to self-care correlated with all the study variables with the exception of the level of education, self-perceived severity, worry and fear of hypoglycemia, concern about complications, diabetes related-distress, and the ViDa1 worry subscale (see Figure 1).

### 5.3. Predictors of Self-Care in Type 1 Diabetes

The results of the multivariate analysis are shown in Table 3. The set of four predictor variables accounted for 32% of the variance of the self-care variable (*R*^2^ = 0.32). High scores for level of motivation, level of training in diabetes management, importance given to the illness, and self-efficacy were associated with higher scores for adherence to self-care behaviors.

## 6. Discussion

The main contribution of this study is the identification of psychosocial factors which determine the extent to which people with type 1 diabetes engage with their health care. Motivation, training in diabetes management, beliefs regarding the disease, and self-efficacy were the main contributors to adherence to self-care behaviors.

Although this is a topic that has been widely discussed in the literature [8, 11, 26], to our knowledge, there are no studies that have assessed these variables together or considered their possible interrelations. This multicenter study assesses personality variables, emotional state, motivation, family and social support, and belief systems in a wide and heterogeneous sample of patients.

Our results suggest that motivation is the most important factor in self-care behaviors: the more motivated the patients are with regard to their health care, the greater their adherence. Indeed, prior interventions with motivational interviewing, aimed at improving motivation, have shown improvements in glycemic control in adolescents with type 1 diabetes [27–29].

The level of training in diabetes management is another important factor. Patients in this study have a high self-perceived level of training, which facilitates the management of their diabetes and increases adherence to self-care.

Beliefs regarding health may also affect the extent to which people engage in the care of their disease. Patients who have a greater awareness of their disease and attribute importance to it present higher levels of adherence to self-care and better glycemic control, as do patients who are particularly concerned about developing hypoglycemia or chronic complications. Indeed, the lack of awareness may be an underlying factor affecting self-care attitudes and practices [30]. Previous studies have supported the role of beliefs in self-care behaviors in diabetes [31, 32] and in heart failure [33].

As for self-efficacy, we found an association between higher scores in this variable and greater adherence, in agreement with earlier studies in type 1 diabetes [14, 34, 35]. Although its contribution to the explanatory model of adhesion is small, self-efficacy is closely related to training and motivation and may have a mediating effect. Self-reliance is the key to implementing what one has learned and also facilitates decision-making. In fact, it has proven to be a predictor of response to motivational interviewing [27]. Other personality variables such as conscientiousness, PA, and NA showed weak correlations in the bivariate analysis and did not enter the multivariate analysis as significant factors. Previous studies have associated conscientiousness [36] and PA [37, 38] with glycemic control.

In our study, although patients reported a high level of training in diabetes management, more than half reported having difficulty following treatment recommendations. This was not only due to a lack of motivation, but also because of problems related to their emotional state. Using the established cutoffs, the prevalence on anxiety and depression was high in our patients (57.1% and 23.1%, respectively), more than double the prevalence found in background population [39] and similar to [40] or higher than [41] other studies performed in people with type 1 diabetes [42]. In agreement with other authors, people with high scores on anxiety [40] and depression [43, 44] had lower adherence to self-care behaviors. However, in disagreement with a previous report, we found no significant correlation between diabetes-related distress (PAID) and adherence to self-care behaviors [45]. Emotional state proved to be an obstacle to the effectiveness of an educational intervention in patients with heart failure [46], and a reduction in depressive symptoms was associated with improved metabolic control in type 1 diabetes [43].

Negative emotional status can be a major barrier and can interfere with motivation for self-care. Cognitive behavioral therapy (CBT) has been shown to improve adherence and depression and glycemic control and raise levels of self-care in patients with type 2 diabetes [47] and has also shown to reduce depressive symptoms and diabetes-related anxiety in adults with type 1 diabetes [48].

In the present study, adherence to self-care was associated with more social support, in agreement with previous evidence [14, 49] and with health-related quality of life [50, 51].

To identify the factors associated with adherence to self-care behaviors is very relevant in type 1 diabetes. Indeed, the American Diabetes Association [52] recommends assessing patients' emotional state, health-related quality of life, and disease-related beliefs in their routine visits. This assessment can be used to design strategies that approach these aspects. In fact, interventions that consider several factors simultaneously, such as emotional, social, and family issues, have proven to be more efficacious in improving adherence than single approaches [53].

We are aware that our study has some limitations. Some variables such as motivation and fear of hypoglycemia were measured with a single item, although the prevalence of fear of hypoglycemia was similar to that reported by other studies which used the FH-15 scale [54]. In addition, it was the patients themselves who reported their level of training, as well as other issues. This is a subjective impression and patients may or may not have provided sincere answers. The presence of a researcher during the completion of the questionnaires assured that the patients themselves completed them and allowed for clarifications to be made if needed. Finally, we did not take into account the patients' economic situation, but we are aware that this can influence self-care behaviors. However, our study was aimed at identifying factors that might be modified in clinical practice.

## 7. Conclusion

In the present study assessing patients with type 1 diabetes, motivation, training in diabetes management, beliefs regarding the disease, and self-efficacy were the main contributors to adherence to self-care behaviors. On the other hand, anxiety and depression were highly prevalent and associated with lower adherence.

## Figures and Tables

**Table 1:** Participants' characteristics. *N* = 428.

Sex (% women)	58
Age (years)ª	36 (11.8)
Duration of disease (years)ª	18.3 (10.2)
HbA1c (%)ª	7.9 ± 1.3%
HbA1c (mmol/mol)ª	63 ± 14.2
Insulin treatment (%)	
Multiple injections	78.1
Pump	18.4
Carbohydrate counting (%)	67.5
At least one event (% of patients)	
Mild hypoglycemia (weekly)	89.6
Severe hypoglycemia (any time)	31.1
Admission due to hyperglycemia (any time)	28.3
Drug treatment for depression or anxiety (%)	16
Cardiovascular risk factors (%)	41.7
Sedentary (%)	32.5
Complications (%)	30.9
Retinopathy	24.1
Nephropathy	9.2
Neuropathy	12.3
Macroangiopathy	3.5
Limited by complications (%)	9
Lives with (%)	
Family	71.9
Partner	18.6
Alone	7.5
Other	2
Education (%)	
Unqualified	1.2
Primary	30.4
Secondary	38.4
University	30
Occupation (%)	
Student	14.9
Employed	55.7
Unemployed	22.6
Other	6.9

ªData are expressed as mean (*SD*).

**Table 2:** Means (SD), minimum and maximum scores, scales, and Cronbach's alpha for variables (*N* = 428).

	Minimum	Maximum	Mean (SD)	Score	Cronbach's alpha
Level of training in diabetes management	1	10	7.3 (1.6)	0-10	
Level of motivation for self-care	1	10	6.6 (2.4)	1-10	
Level of awareness of the disease	0	10	8.6 (1.6)	0-10	
Level of importance given to the disease	0	10	8.3 (1.8)	0-10	
Level of perceived severity	0	10	7.2 (2.2)	0-10	
Self-efficacy	15	50	37.2 (6.7)	1-50	0.89
Conscientiousness	11	35	26.2 (4.8)	7-35	0.69
Positive affect	0	30	18.9 (6.3)	0-30	0.86
Negative affect	0	30	11.1 (6.7)	0-30	0.78
Anxiety	1	21	8.4 (3.0)	0-21	0.55
Depression	0	17	4.7 (3.7)	0-21	0.77
Diabetes-related distress	0	80	37.5 (18.9)	0-100	0.94
Fear of hypoglycemia	1	5	3.5 (1.3)	1-5	
Concern about hypoglycemia	1	5	3.7 (1.1)	1-5	
Concern about complications	1	5	4.1 (1.0)	1-5	
Interference of diabetes (ViDa1)	12	57	29 (9.8)	12-60	0.86
Self-care (ViDa1)	15	55	41.6 (7.9)	11-55	0.84
Well-being (ViDa1)	8	30	22.5 (5.1)	6-30	0.76
Worry about the disease (ViDa1)	5	25	18.9 (4.1)	5-25	0.70
Social support	34	95	83.7 (12.5)	19-95	0.95
Self-care (SCI-R)	8.3	100	65.4 (15.1)	0-100	0.79

**Table 3:** Stepwise multiple linear regression analysis with dependent variable self-care (SCI-R) and the independent variables (*N* = 428).

Predictors	*B*	Standard error	Beta	*t*	Sig.	*F*	*R* ^2^
*Model 1*							
Motivation for self-care	3.17	0.25	0.51	12.4	<0.001	155.70 (1,422)	0.26
*Model 2*							
Motivation for self-care	2.86	0.26	0.46	10.89	<0.001	88.09 (2,421)	0.29
Level of training in diabetes	1.56	0.40	0.16	3.90	<0.001		
*Model 3*							
Motivation for self-care	2.36	0.29	0.38	8.09	<0.001	64.82 (3,420)	0.31
Level of training in diabetes	1.49	0.39	0.16	3.77	<0.001		
Level of importance given to disease	1.37	0.38	0.16	3.62	<0.001		
*Model 4*							
Motivation for self-care	2.21	0.29	0.36	7.48	<0.001	51.36 (4,419)	0.32
Level of training in diabetes	1.44	0.39	0.15	3.68	<0.001		
Level of importance given to disease	1.28	0.37	0.15	3.39	0.001		
Self-efficacy	0.26	0.09	0.11	2.80	0.005		

## Data Availability

The data included in this study are in paper format in our hospital and in digital format on an electronic database.
